# Successful Combination of the Slipstream and the Tip Detection–Antegrade Dissection and Re-Entry Techniques for Chronic Total Occlusion

**DOI:** 10.1016/j.jaccas.2025.105669

**Published:** 2025-10-19

**Authors:** Masataka Yoshinaga, Wakaya Fujiwara, Kenya Nasu, Takashi Muramatsu, Akane Miyazaki, Makoto Fujioka, Takehiro Ito, Masato Ishikawa, Yoshihiro Sobue, Eiichi Watanabe

**Affiliations:** a Department of Cardiology, Fujita Health University Bantane Hospital, Aichi, Japan; b Department of Cardiology, Mie Heart Center, Mie, Japan; c Department of Cardiology, Fujita Health University Hospital, Aichi, Japan; d Department of Clinical Engineering, Fujita Health University Bantane Hospital, Aichi, Japan

**Keywords:** bailout, chronic total occlusion, imaging, intravascular ultrasound, slipstream, tip detection method

## Abstract

**Objective:**

We describe combining the slipstream technique with the tip detection–antegrade dissection and re-entry (TD-ADR) technique using AnteOwl intravascular ultrasound (IVUS) for challenging calcified chronic total occlusion lesions when conventional IVUS-guided approaches fail.

**Key Steps:**

The procedure included the following steps. 1) Confirm on IVUS that the first guidewire is in the subintimal space. 2) Re-insert the AnteOwl IVUS using the first wire lumen only. 3) Advance a Sasuke double-lumen catheter over the same wire to the IVUS tip. 4) Introduce a second guidewire (Conquest Pro 12 Sharpened Tip with 15° bend) through the second wire port. 5) Perform TD-ADR under IVUS guidance to achieve true lumen re-entry.

**Potential Pitfalls:**

IVUS cannot directly visualize the advancement of the microcatheter over the true lumen second guidewire, thus careful fluoroscopic guidance is crucial.

**Take-Home Messages:**

This combined approach provides an effective bailout strategy when microcatheter advancement fails owing to severe calcification. The dual-lumen design, real-time pullback system, and short tip-to-transducer distance of the AnteOwl IVUS optimizes both the slipstream and TD-ADR techniques simultaneously.

## Visual Summary

**Figure:**
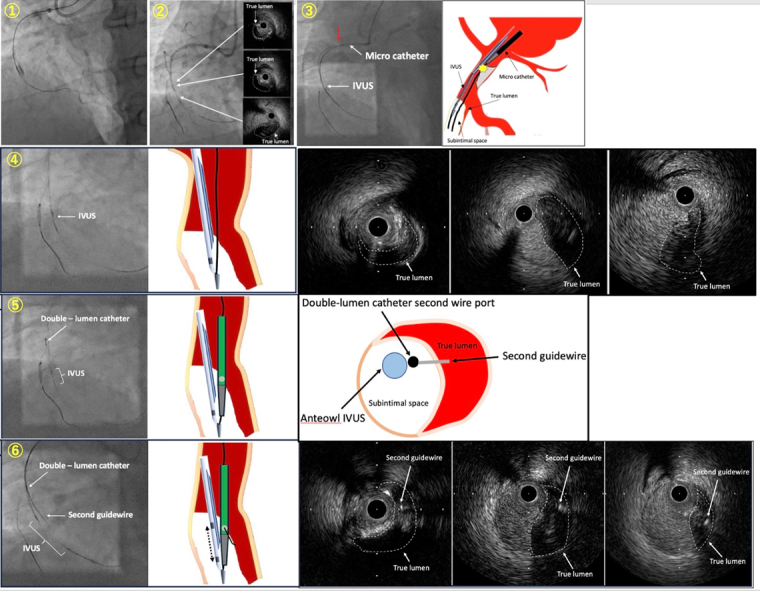

Percutaneous coronary intervention (PCI) for chronic total occlusion (CTO) lesions remains technically challenging despite significant advancements in procedural techniques and devices. Both retrograde and conventional antegrade approaches, particularly those using intravascular ultrasound (IVUS) guidance, have greatly improved procedural success rates.^1^ Several IVUS-guided techniques have been proposed from Japan, including the slipstream technique, first reported by Kinoshita et al.^2^ In this method, only the first guidewire lumen of the Navifocus WR IVUS (Terumo) is used for insertion, after which a double-lumen catheter is advanced along the same guidewire used for the IVUS catheter, facilitating re-entry into the true lumen.^2^ On the other hand, the tip-detection method by Okamura et al^3^, ^4^ and the tip detection–antegrade dissection and re-entry (TD-ADR) technique by Tanaka et al^3^, ^4^ use the AnteOwl IVUS (AO-IVUS) (Terumo) to precisely guide wire manipulation within the subintimal space. The tip-detection method is also recommended in the algorithm of the Asia Pacific Chronic Total Occlusion Club.^1^

**Take-Home Messages**

• Combining the slipstream technique and tip-detection method using AnteOwl IVUS can enhance the stability and accuracy of guidewire manipulation, potentially making re-entry from the subintimal space into the true lumen easier during complex CTO-PCI procedures.
• This technique may serve as a useful option for PCI operators to consider when conventional CTO-PCI approaches prove challenging.

A common feature of these methods is the need to insert the IVUS catheter into the subintimal space at least once.^2^, ^3^, ^4^, ^5^, ^6^, ^7^ However, the conventional tip-detection method proposes the use of IVUS along with an additional wire and microcatheter, which can be limited by the inability to advance the catheter, especially in cases of severe calcification or tortuous lesions. To overcome these limitations, we performed the slipstream technique using AO-IVUS. The AO-IVUS, similar to the Navifocus, features an integrated dual-lumen structure, but in addition, the length from the tip to the transducer has been reduced, and a newly installed real-time pullback system enables precise observation of the target true lumen, which is essential for both methods. Thus, the AO-IVUS may be ideally suited for the slipstream technique. To the best of our knowledge, this is the first reported case of successful CTO-PCI using the slipstream and tip-detection methods in combination with the AO-IVUS catheter after failure of the conventional tip-detection approach.

## Case Summary

**Figure 1:**
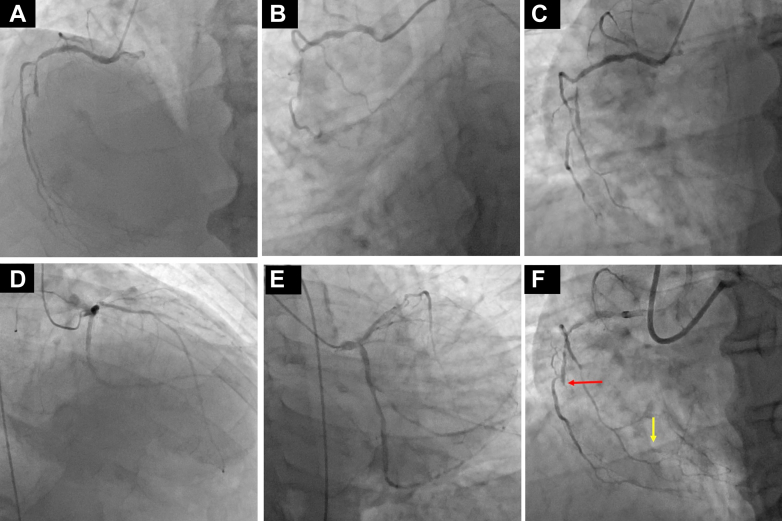
Previous Procedure Information (A) Right coronary angiography (LAO 30°). (B) Right coronary angiography (LAO 30°/CAU 35°). (C) Right coronary angiography (LAO 36°/CRA 37°). (D) Left coronary angiography (RAO 31°/CRA 29°). (E) Left coronary angiography (CAU 34°). (F) Right coronary angiography (LAO 30°) showing CTO entrance (red arrow) and CTO exit (yellow arrow). CAU = caudal; CRA = cranial; LAO = left anterior oblique; RAO = right anterior oblique.

A 63-year-old man presented with Canadian Cardiovascular Society class III angina. He was 169 cm tall, weighed 81.5 kg (body mass index: 28.5), and had a blood pressure of 163/90 mm Hg with a heart rate of 67 beats/min. He had been on hemodialysis for 20 years. Echocardiography showed a reduced left ventricular ejection fraction of 33% with severe hypokinesis from the lateral to the posterior wall. Coronary angiography revealed a CTO lesion approximately 60 mm in length in the right coronary artery (RCA), with a Japanese Retrograde CTO score of 4 (Figures 1A to 1E). After undergoing PCI to the left circumflex artery, the patient opted for PCI to the RCA-CTO. Given the impaired cardiac function and severe calcified stenosis in the donor left anterior descending artery, a retrograde approach posed high ischemic risks. Therefore, we initially planned an antegrade approach with IVUS-guided wiring as a bailout strategy in case of guidewire entry into the subintimal space.

## Procedural Steps

**Figure 2:**
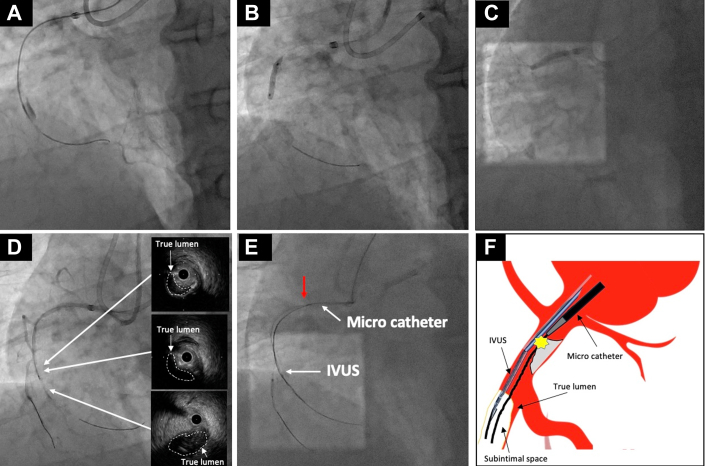
Percutaneous Coronary Intervention (A) In the antegrade approach, neither a Gaia Next2 wire nor a Gladius wire were able to pass through the CTO lesion using angiography. (B and C) POBA was performed for the proximal lesion using a Kizashi 3.0 × 15 mm balloon. (D) We confirmed that the Gladius wire was in the subintimal space distal to the CTO entrance. (E and F) Neither the Corsair-Pro nor the Caravel microcatheter were able to pass through the calcified lesion cracked by POBA near the RCA ostium (red arrow). Fluoroscopy was performed with the adjunctive use of the “SPOT ROI” function^8^ available on the Alphenix Evolve Edition x-ray system (Canon Medical Systems). CTO = chronic total occlusion; POBA = plain-old balloon angioplasty; RCA = right coronary artery; ROI = region of interest.

For the antegrade approach, an 8-F Amplatz left 1.5 guiding catheter was inserted into the RCA via the right femoral artery, and for the retrograde approach, a 7-F SPB 3.5 guiding catheter was inserted into the left coronary artery via the left femoral artery. In the antegrade approach, neither a Gaia Next2 wire (Asahi Intecc) nor a Gladius wire (Asahi Intecc) supported by a Corsair-Pro microcatheter (Asahi Intecc) were able to pass through the CTO lesion using angiography (Figure 2A). The Gladius wire (first guidewire) was left in the subintimal space approximately 4 cm distal from the CTO entry, and the Corsair-Pro microcatheter was advanced about 2 cm from the entry to secure space for IVUS catheter insertion. Next, we attempted to advance an AO-IVUS over the Gladius wire, but the AO-IVUS could not pass owing to severe calcified stenosis in the proximal segment. Therefore, plain-old balloon angioplasty (POBA) was performed on the proximal calcified lesion using a 15-mm Kizashi 3.0 × balloon (Kaneka) (Figures 2B and 2C). Subsequently, by using the anchor balloon technique with a Ryurei 2.0 × 10 mm balloon (Terumo), we were able to advance the AO-IVUS along the Gladius wire and confirm that the Gladius wire was in the subintimal space distal to the CTO entrance (Figure 2D, Video 1).

**Figure 3:**
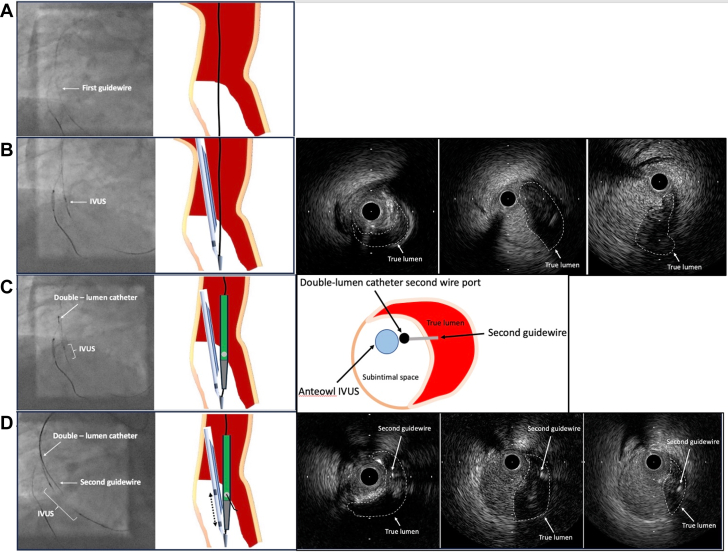
Schematic Diagram of Procedural Steps for the Combination Slipstream and TD-ADR Technique (A) After removing the IVUS once, (B) we reinserted it using only the first (Gladius) guidewire lumen and confirmed the first guidewire and IVUS at the subintimal space with IVUS. (C) We advanced the Sasuke double-lumen catheter over the same Gladius wire to the tip of the IVUS catheter. (D) Subsequently, the CP12ST guidewire was advanced through the second wire port of the Sasuke double-lumen catheter. By creating a secondary bend of approximately 15° on the CP12ST, TD-ADR was successfully performed, enabling accurate guidewire advancement into the true lumen. Fluoroscopy was performed with the adjunctive use of the “SPOT ROI” function^8^ on the Alphenix Evolve Edition x-ray system (Canon Medical Systems). CP12ST = Conquest Pro 12 Sharpened Tip; IVUS = intravascular ultrasound; ROI = region of interest; TD-ADR = tip detection–antegrade dissection and re-entry.

To perform the conventional tip-detection method, we placed a Conquest Pro 12 Sharpened Tip (CP12ST) (Asahi Intecc) (second antegrade wire) in the same subintimal space using a Sasuke double-lumen catheter (Asahi Intecc) and attempted to advance a Corsair-Pro microcatheter over the wire, however neither the Corsair-Pro nor the Caravel microcatheter (Asahi Intecc) were able to cross the calcified lesion despite prior POBA cracking near the RCA ostium (Figures 2E and 2F). Although stenting was considered, it was abandoned owing to the proximity to the ostium and concerns about potential stent fracture caused by the guide catheter. As this process was very time consuming, we decided to perform the slipstream technique^2^ (Video 2) using AO-IVUS. The procedural steps are demonstrated in Figures 3A to 3G. After removing the IVUS once (Figure 3A), we reinserted it using only the first guidewire lumen (Figure 3B) and advanced the Sasuke double-lumen catheter over the same Gladius wire to the tip of the IVUS catheter (Figure 3C, Video 3). Subsequently, the CP12ST was advanced through the second wire port of the Sasuke double-lumen catheter. But intraplaque tracking using the tip-detection method was difficult because of severe calcification at the entrance. Therefore, by creating a secondary bend of approximately 15° on the CP12ST, TD-ADR was performed, allowing precise advancement of the second antegrade guidewire into the true lumen (Figure 3D, Video 4).

**Figure 4:**
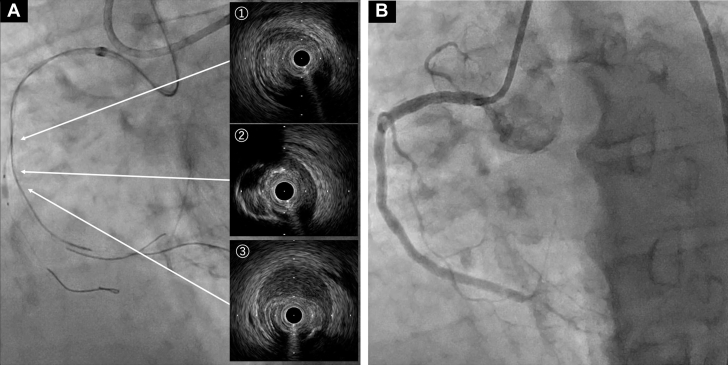
Prestent IVUS and Final Angiography (A) The Gladius wire was able to directly cross the CTO exit. AnteOwl IVUS revealed Gladius wire progression: (①) true lumen proximal to CTO, (②) subintimal space immediately after CTO entry, (③) re-entry into true lumen approximately 1 cm distal to CTO entry. (B) Final angiography image. CTO = chronic total occlusion; IVUS = intravascular ultrasound.

Afterward, we angiographically confirmed the location where the tip of the wire was definitely in the true lumen and attempted to advance the Corsair-Pro microcatheter to that point, but we were unable to cross the heavily calcified CTO entrance. By switching to a Corsair-XS microcatheter (Asahi Intecc), we successfully advanced the microcatheter to the angiographically confirmed true lumen. Thereafter, the Gladius wire was able to directly cross the CTO exit, and AO-IVUS observation revealed that the wire had entered the true lumen 1 cm beyond the CTO entrance (Figure 4A, Video 5). The CTO lesion was dilated with 3 drug-eluting stents, and normal antegrade blood flow was achieved (Figure 4B).

The patient was discharged the following day without complications.

## Potential Pitfalls

Careful attention is essential during removal of the double-lumen catheter to prevent inadvertent withdrawal of the wire from the true lumen. Furthermore, since IVUS imaging cannot directly visualize subsequent microcatheter insertion over the true lumen wire, cautious fluoroscopic guidance is necessary throughout this step to ensure procedural safety.

## Discussion

In conventional IVUS-guided approaches, it is generally necessary to advance both the IVUS catheter and an additional microcatheter simultaneously. This technique tends to be time consuming and can complicate the procedure, especially when device passage through severely calcified lesions is difficult. This case suggests that the combination of the slipstream technique and tip-detection method using AnteOwl IVUS can be clinically useful and feasible. It has also been reported that double-lumen catheters provide greater stability within the vessel compared with conventional microcatheters during guidewire manipulation.^2^ In our case, we selected the Sasuke double-lumen catheter, which has a thinner and more lubricious tip than the Crusade and is identical to that of the Corsair, potentially reducing the risk of vascular injury. Regarding the choice of IVUS catheter, when it is necessary to puncture from the subintimal space into the true lumen, the IVUS catheter itself must first be advanced into the subintimal space.^2^, ^3^, ^4^, ^5^, ^6^, ^7^ To minimize vascular injury, it is important to insert the catheter as short a distance as possible. Therefore, an IVUS catheter with the shortest tip-to-transducer distance and lowest shaft profile is optimal for this technique. The AO-IVUS catheter, with its dual-lumen structure, shortened tip-to-transducer distance (8 mm), reduced shaft diameter (3.1-F), and real-time pullback imaging capability, can overcome these limitations, allowing for more precise navigation and re-entry into the true lumen. Thus, compared with the Navifocus (tip-to-transducer distance: 9 mm, shaft diameter: 3.2-F) or Opticross (tip-to-transducer distance: 20 mm, shaft diameter: 3.0-F) (Boston Scientific), the AO-IVUS may be more suitable and advantageous for the slipstream technique. On the other hand, compared with the standard use of AO-IVUS via the second guidewire lumen, the pushability in this approach is inferior, potentially resulting in decreased crossability. Although the use of an 8-F guiding catheter is generally recommended for these techniques, our experience suggests that this strategy is sufficiently feasible even with a 7-F guiding catheter.

This case is an important example demonstrating improved procedural efficiency through the combination of the slipstream technique and TD-ADR using AO-IVUS. This technique can enhance the stability and accuracy of guidewire manipulation, potentially facilitating easier TD-ADR. Where AO-IVUS is not available, the technique can be reproduced using an IVUS with a short tip-to-transducer distance and reliable pullback, and where CP12ST is not available, any penetration-grade tapered wire may be substituted, maintaining the core concept and procedural feasibility. This novel combination potentially broadens procedural capabilities in complex CTO lesions, suggesting applicability in a wider range of challenging cases. Further validation in case series or registries is warranted.

## Conclusions

The combination of the slipstream and tip-detection techniques using AO-IVUS provides an effective alternative strategy for treating challenging CTO lesions that are resistant to conventional IVUS-guided methods. This approach is a valuable option for challenging and complex CTO lesions.

**Visual Summary:**
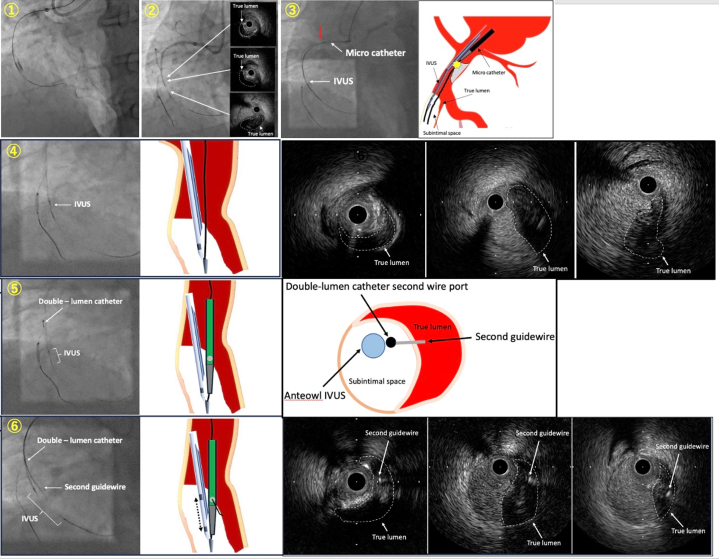
Novel Slipstream and Tip Detection Technique for Efficient and Precise ADR IVUS = intravascular ultrasound.

## Funding Support and Author Disclosures

The authors have reported that they have no relationships relevant to the contents of this paper to disclose.

## Appendix

Video 1 IVUS Demonstrating Initial Guidewire Located in the Subintimal Space Distal to CTO Entry

Video 2 This Is an Ex Vivo Experiment In this system, the second guidewire port of the Sasuke double-lumen catheter is located near the transducer of the AnteOwl IVUS, allowing the operator to support the wire through the second port and precisely manipulate it under IVUS imaging. By moving the probe back and forth from just before the entry, through the transition from the true lumen to the subintimal space, and toward the distal segment, the tip-detection method can be performed, potentially enabling accurate advancement of the second guidewire into the true lumen.

Video 3 Angiography Confirmation of Reinserted AnteOwl IVUS Using Only the First Guidewire Lumen and Advancement of the Sasuke Double-Lumen Catheter Over the Same Gladius Wire to the Tip of the IVUS Catheter

Video 4 IVUS Confirmation Showing the Successful Advancement of the CP12ST Guidewire Into the True Lumen via TD-ADR

Video 5 IVUS Demonstrating Re-entry of the Gladius Guidewire Into the True Lumen Distal to CTO Entry
